# Analysis of Patients With Monocytic and Monocytic‐Like Acute Myeloid Leukemia, Including AML‐M4 and AML‐M5, Treated With Venetoclax Plus Azacitidine

**DOI:** 10.1002/ajh.70161

**Published:** 2026-01-05

**Authors:** Marina Konopleva, Courtney D. DiNardo, Yan Sun, Paul Jung, Sanam Loghavi, Jalaja Potluri, Monique Dail, Brenda Chyla, Daniel A. Pollyea

**Affiliations:** ^1^ Department of Oncology Montefiore Einstein Cancer Center The Bronx New York USA; ^2^ Department of Leukemia The University of Texas MD Anderson Cancer Center Houston Texas USA; ^3^ AbbVie Inc. North Chicago Illinois USA; ^4^ Department of Hematopathology The University of Texas MD Anderson Cancer Center Houston Texas USA; ^5^ Division of Genentech Inc. South San Francisco California USA; ^6^ Division of Hematology, School of Medicine University of Colorado Aurora Colorado USA

To the Editor,

1

Acute myeloid leukemia (AML) is a heterogeneous malignancy with variable outcomes to treatment. Frontline therapy typically consists of high‐dose chemotherapy followed by stem cell transplant for patients who are able to tolerate high‐intensity treatment, or low‐dose chemotherapy (e.g., cytarabine or hypomethylating agents, like azacitidine) for patients who are older and/or have comorbid conditions, although the exact treatment course used for a given patient depends upon disease biology and evolving research. Venetoclax‐azacitidine increased response rates versus azacitidine monotherapy among patients with AML who are ineligible for intensive chemotherapy [1], leading to Food and Drug Administration approval in 2018 and has since become the standard of care for this patient population. Venetoclax‐azacitidine has shown broad efficacy across patient subgroups, including those with primary or secondary AML, intermediate or poor cytogenetic risk, and mutation subgroups (e.g., *IDH1/2*‐mutated AML treated with or without IDH inhibitor) [1, 2, 3]. However, patients with monocytic AML have been reported to have primary and secondary resistance to and/or suboptimal response with venetoclax‐based therapy [4]. In a study of 100 patients, those with French–American–British (FAB) M5 AML subtype [5], a more differentiated phenotype of monocytic AML, were suggested to be less sensitive to treatment with venetoclax‐azacitidine [6]. Other studies, both in vivo and ex vivo, have shown similar results [4, 7, 8]. An emerging 4‐gene prognostic signature for AML highlights the influence of mutations in *TP53*, *FLT3‐*ITD, *NRAS*, and *KRAS* on patient outcomes, of which *N/KRAS* mutations are commonly associated with monocytic AML [9, 10]. Here, we report findings by AML differentiation state using the FAB classification system (M4, M5) and baseline gene expression profiling (GEP) to define monocytic‐like AML in a post hoc analysis of venetoclax‐azacitidine in patients ineligible for intensive chemotherapy from a pooled analysis of Phase 1b M14‐358 and Phase 3 VIALE‐A studies.

Patients from the Phase 1b M14‐358 (NCT02203773) and Phase 3 VIALE‐A (NCT02993523) studies [1, 11] who received venetoclax‐azacitidine were included (Figure S1, Tables S1 and S2). Two methods were used to define monocytic AML: pathologic assignment of FAB subtyping (M4, M5, non‐M4/M5; *n* = 197) per investigator and baseline GEP in patients with > 30% AML blasts (*n* = 153). Seventy‐seven patients had FAB and GEP data. For GEP, a 13‐gene panel of common myeloid markers (*ANPEP*, *CD14*, *CD300e*, *CD33*, *CD34*, *CD4*, *CD68*, *CR1*, *FCGR1A*, *FCGR1B*, *FCGR1CP*, *ITGAM*, and *KIT*) was used (Figure 1A). The expression levels of 4 of these genes associated with monocytic differentiation (*CD14*, *ITGAM* [*CD11b*], *CD300e*, and *CR1* [*CD35*]) were ultimately used as a signature to classify patients as monocytic or monocytic‐like AML (above) or non‐monocytic AML (below), based on being above/below the median GEP signature value. Monocytic markers based on European LeukemiaNet (ELN) guidelines (*CD14*, *CD36*, *CD64*, *CD4*, *CD38*, and *CD11c*) were also analyzed [12]. Complete remission (CR) and CR with incomplete marrow recovery (CRi) rates and median overall survival (OS) were assessed. RNA sequencing and mutational profiling using the MyAML targeted panel were performed on baseline bone marrow aspiration specimens. Gene expression of BCL‐2 family members was determined for individual mutation subgroups.

**FIGURE 1:**
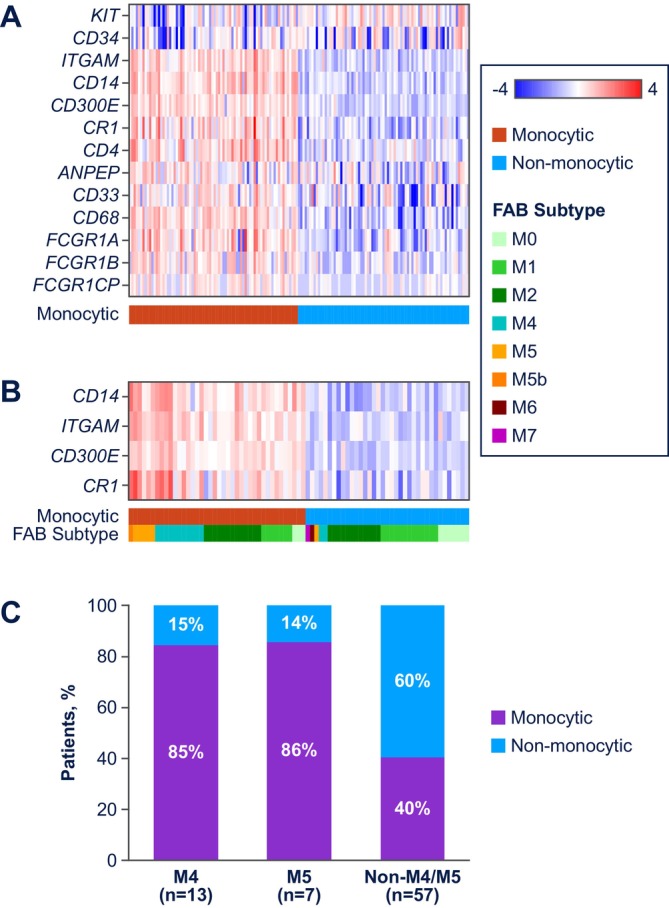
AML differentiation state using FAB and GEP classification. (A) Heatmap of expanded panel of common myeloid markers dysregulated in AML, (B) heatmap of GEP signature, and (C) FAB versus monocytic concordance. AML, acute myeloid leukemia; FAB, French–American–British classification.

Primary outcomes from the Phase 1b M14‐358 and Phase 3 VIALE‐A studies have been previously reported [1, 11]. According to FAB subtyping in the current analysis, 32, 24, and 141 patients had M4, M5, or non‐M4/M5 AML, respectively (Figure S1). Using GEP, 76 and 77 patients were categorized with monocytic AML or non‐monocytic AML, respectively. Concordance between FAB and GEP classification is shown in Figure 1B,C. Among the FAB evaluable samples, 86% (6/7) with M5 and 85% (11/13) with M4 were identified as monocytic AML (Figure 1C).

Clinical outcomes in monocytic versus non‐monocytic FAB subtypes were similar to those in monocytic versus non‐monocytic GEP subtypes (Figure S2, Figure 2). By FAB classification, CR + CRi rates (n/N; 95% CI) were 63% (20/32; 45.2–77.1) for M4, 58% (14/24; 38.8–75.5) for M5, and 71% (100/141; 62.9–77.8) for non‐M4/M5 AML. By GEP subtype, CR + CRi rates were 62% (47/76; 50.6–71.9) for monocytic AML and 69% (53/77; 57.8–78.1) for non‐monocytic AML. By FAB, median OS (95% CI) was 12.4 months (3.4–32.5) for M4, 16.8 months (5.8–27.5) for M5, and 14.7 months (10.7–22.3) for non‐M4/M5. For GEP, median OS was 14.7 months (8.2–24.3) for monocytic AML and 15.2 months (10.6–20.5) for non‐monocytic AML. Clinical outcomes in GEP subtypes using the 6‐gene panel based on ELN guidelines were similar to results with the 4‐gene panel noted above (Figure S3). To control for features of the monocytic gene signature, the top quartiles of the GEP data were evaluated. Patients in the top quartile of the GEP signature had similar median OS compared with the overall results for monocytic AML and non‐monocytic AML (Figure S4).

**FIGURE 2:**
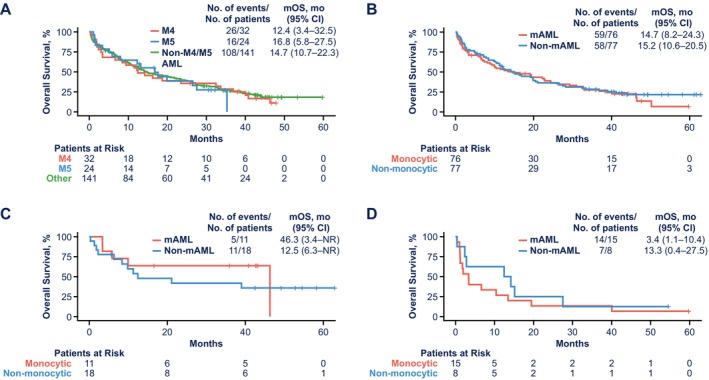
OS for patients treated with venetoclax‐azacitidine by (A) FAB subtype and (B) by GEP subtype and OS in patients with GEP‐defined monocytic versus non‐monocytic AML status and (C) *NPM1* mutation and (D) *N/KRAS* mutation. AML, acute myeloid leukemia; FAB, French–American–British classification; GEP, gene expression profiling; mAML, monocytic AML; mOS, median overall survival; NR, not reached; OS, overall survival.

Mutation analysis was performed for patients with M4 (*n* = 24), M5 (*n* = 13), and non‐M4/M5 (*n* = 108) AML. Among patients with *NPM1* mutation (*n* = 22), 14% (3/22) were M4, 18% (4/22) were M5, and 68% (15/22) were non‐M4/M5. Among patients with *N/KRAS* mutation (*n* = 24), 33% (8/24) were M4, 17% (4/24) were M5, and 50% (12/24) were non‐M4/M5 (Table S3). In monocytic versus non‐monocytic AML, *NPM1* mutation rates were 15% versus 24% and *N/KRAS* mutation rates were 20% versus 11%, respectively (Table S4). Overlapping *NPM1* and *N/KRAS* mutations are presented in Table S5. Importantly, molecular characteristics and/or molecular drivers in combination with differentiation state were more predictive of survival outcomes than differentiation state alone (Figure 2). In the monocytic AML group by GEP, among 11 patients with *NPM1* mutation, the CR + CRi rate was 64% (35.4–84.8) and median OS was 46.3 months (3.4‐not reached) (Table S4, Figure 2). In patients with monocytic AML and *NPM1* wild‐type, the CR + CRi rate was 60% (48.0–71.5) and median OS was 11.5 months (6.6–22.3) (Figure S5A). In the non‐monocytic AML group, among 18 patients with *NPM1* mutation, the CR + CRi rate was 72% (49.1–87.5) and median OS was 12.5 months (6.3‐not reached). *NPM1*‐mutated monocytic AML was associated with more favorable clinical outcomes compared with *N/KRAS*‐mutated monocytic AML. In patients with *N/KRAS‐*mutated monocytic AML (*n* = 15), the CR + CRi rate was 33% (15.2–58.3) and median OS was 3.4 months (1.1–10.4) (Table S4, Figure 2). In patients with *N/KRAS*‐mutated non‐monocytic AML (*n* = 8), the CR + CRi rate was 75% (40.9–92.8) and median OS was 13.3 months (0.4–27.5). Clinical outcomes in patients without *NPM1* or *N/KRAS* mutations in the monocytic and non‐monocytic AML groups are presented in Table S4 and Figure S5. BCL‐2 family expression across monocytic versus non‐monocytic AML and *MCL1* gene expression by mutation type in monocytic versus non‐monocytic AML is shown in Figure S6. Among patients with mutated *NPM1* and *N/KRAS*, the expression of *BCL2* was lower and *BCL2A1* was higher in monocytic AML compared with non‐monocytic AML.

Response rates with venetoclax‐azacitidine treatment were 62% and 58% for M4 and M5 AML, respectively, and 63% for GEP‐defined monocytic AML, slightly lower than response rates for non‐M4/M5 (71%) and GEP‐defined non‐monocytic AML (69%). These results are aligned with previous studies that described worse outcomes for patients with monocytic AML treated with venetoclax‐based therapy, including venetoclax‐azacitidine‐refractory disease, finding lower response rates and significantly shorter median OS compared with patients with a non‐monocytic phenotype [4, 6, 7, 8]. In contrast with previous studies, median OS was similar across all groups: 12.4 and 16.8 months for M4 and M5 AML, respectively, and 14.7 months for GEP‐defined monocytic AML, compared with 14.7 months for non‐M4/M5 AML and 15.2 months for GEP‐defined non‐monocytic AML.

Differences in response and survival among patients with monocytic AML were associated with the presence or absence of prognostically relevant mutations, including *NPM1* and *N/KRAS* mutations. Patients with *NPM1*‐mutated monocytic AML had the longest median OS of 46.3 months with venetoclax‐azacitidine treatment, whereas patients with *N/KRAS*‐mutated monocytic AML had a median OS of only 3.4 months. Previous studies have also shown that *NPM1‐*mutated AML favorably affected patient outcomes, whereas *N/KRAS‐*mutated AML negatively affected patient outcomes, although these studies did not investigate monocytic status [9, 10].

Venetoclax‐azacitidine response appears to correlate with developmental stage, with phenotypically primitive AML being more sensitive and monocytic differentiation more resistant to treatment [6]. Resistant monocytic AML was previously characterized by loss of *BCL2* expression, which is targeted by venetoclax, with a shift to reliance on *MCL1* to mediate oxidative phosphorylation and cell survival. Similarly, in this study the expression of *BCL2* was decreased and the expression of *MCL1* was increased in monocytic AML compared with non‐monocytic AML. Although baseline gene expression of *BCL2* family members associated with differentiation state was similar across the select mutations in this study, subtle variations may contribute to outcomes with venetoclax‐azacitidine treatment, especially when combined with the broader mutational impacts. While the expression of BCL‐2 and MCL‐1 proteins within leukemia stem cells is hypothesized to be a likely driver of survival outcomes with venetoclax‐azacitidine treatment [10], it was not measured in this study. Another limitation of the current study was the small sample size, particularly for patients with FAB subtyping and mutation data. These data address an active area of research to characterize treatment response in patients belonging to key subgroups [13]. Additional studies with various triplet therapies are underway in clinical settings.

The results of this study highlight the importance of characterizing monocytic differentiation status and mutations in prognostic genes when selecting treatment for patients with AML who are ineligible for intensive therapy. Although recent progress has been made to improve treatment response and extend survival, further research and new treatment approaches are needed to optimize patient outcomes.

## Funding

Venetoclax is being developed in collaboration between AbbVie and Genentech. AbbVie funded this study and participated in the design, study conduct, analysis, and interpretation of data, as well as the writing, review, and approval of the publication. No honoraria or payments were made for authorship.

## Ethics Statement

Both trials were conducted in accordance with the Declaration of Helsinki and Good Clinical Practice guidelines of the International Council for Harmonization. Both protocols were approved by regional review boards and/or ethics committees. **Trial Registration:** Clinicaltrials.gov: NCT02203773, NCT02993523.

## Consent

Patients from both trials provided written informed consent.

## Conflicts of Interest

Marina Konopleva has served as an Investigator on an AbbVie funded study; has received research funding from AbbVie, Allogene, AstraZeneca, Genentech, Gilead, ImmunoGen, MEI Pharma, Precision, Rafael, Sanofi, and Stemline; has served in an advisory/consultancy role for AbbVie, AstraZeneca, Auxenion, Bakx, Boehringer, Dark Blue Therapeutics, F. Hoffman La‐Roche, Genentech, Gilead, Janssen, Legend, MEI Pharma, Redona, Sanofi, Sellas, Stemline, and Vincerx; has stock options/royalties with Reata Pharmaceutical (IP); and has patents with Novartis, Eli Lilly, Reata Pharmaceutical. Courtney D. DiNardo is supported by the LLS Scholar in Clinical Research Award; has received institutional research funding from AbbVie, Astex, BeiGene, Bristol Myers Squibb, Jazz, Foghorn, Schrodinger, and Servier; has served as a consultant/advisory board member for AbbVie, AstraZeneca, Bristol Myers Squibb, Genentech, Genmab, GSK, Notable Labs, Rigel, Schrodinger, and Servier. Yan Sun, Paul Jung, Jalaja Potluri, and Brenda Chyla are employed with AbbVie and may have stock or other options in AbbVie. Sanam Loghavi has received institutional research funding from Amgen and Astellas; has served as a consultant/advisory board member for AbbVie, Bristol Myers Squibb, Caris, Servier, Daiichi Sankyo, Blueprint Medicine, Arima, and Recordati; and has stock in AbbVie. Monique Dail was employed with Genentech and may have stock or other options. Daniel A. Pollyea is supported by the Leukemia and Lymphoma Society's Career Development Program Scholar in Clinical Research Achievement award, has received research funding from AbbVie, Karyopharm, Teva, and Bristol Myers Squibb, and has served as a consultant/advisory board member for ImmunoGen, Novartis, AstraZeneca, Syros, Kura, Bristol Myers Squibb, Ryvu, AbbVie, Magenta, Qihan, Zentalis, Medivir, HiberCell, LINK, Daiichi Sankyo, Schrödinger, Aptevo, Rigel, Sumitomo Pharma, Adicet, and Gilead.

## Supporting information


**Data S1:** ajh70161‐sup‐0001‐Supinfo.pdf.

## Data Availability

AbbVie is committed to responsible data sharing regarding the clinical trials we sponsor. This includes access to anonymized, individual, and trial‐level data (analysis data sets), as well as other information (e.g., protocols, clinical study reports, synopses, or statistical analysis plans), as long as the trials are not part of an ongoing or planned regulatory submission. These clinical trial data can be requested by any qualified researchers who engage in rigorous, independent, scientific research and will be provided following review and approval of a research proposal, Statistical Analysis Plan (SAP), and execution of a Data Use Agreement (DUA). Data requests can be submitted at any time after approval in the US and Europe and after acceptance of this manuscript for publication. The data will be accessible for 12 months, with possible extensions considered. For more information on the process or to submit a request, visit the following link: https://vivli.org/ourmember/abbvie/ then select “Home”.
