# Supplementary material for: Analysis of Patients With Monocytic and Monocytic‐Like Acute Myeloid Leukemia, Including AML‐M4 and AML‐M5, Treated With Venetoclax Plus Azacitidine
Source: Am J Hematol. 2026 Jan 5;101(3):577–80. doi: 10.1002/ajh.70161 (PMC12869004; doi:10.1002/ajh.70161)
Supplement: Supplementary file 1 — Data S1: ajh70161‐sup‐0001‐Supinfo.pdf. [file AJH-101-577-s001.pdf]

SUPPORTING INFORMATION

Supplemental Figure 1. **Pooled analysis patient disposition.** Aza, azacitidine; BMA, bone marrow aspiration; FAB, French-American-British; GEP, gene expression profiling; mAML, monocytic acute myeloid leukemia; Ph, phase; RNAseq, RNA sequencing; Ven, venetoclax.

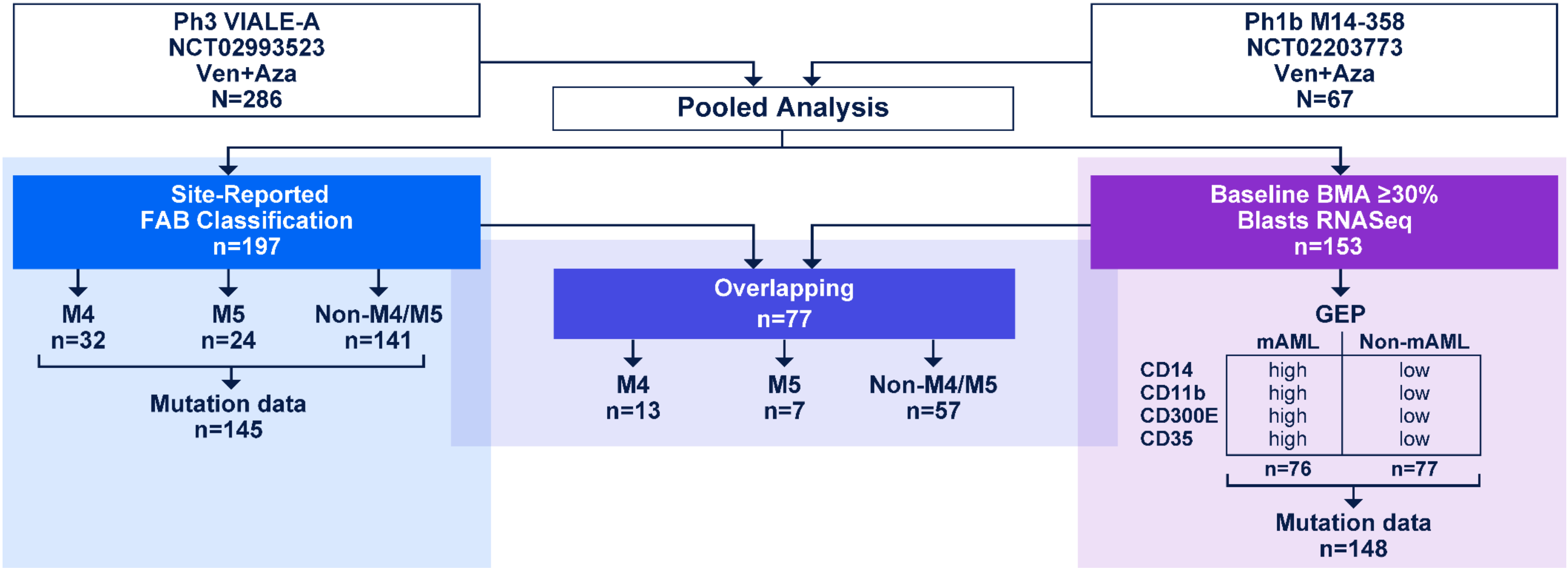

Supplemental Figure 2. CR + CRi rates (A) by FAB subtype and (B) by GEP subtype. CR, complete remission; CRi, complete remission with incomplete marrow recovery; FAB, French-American-British classification; GEP, gene expression profiling.

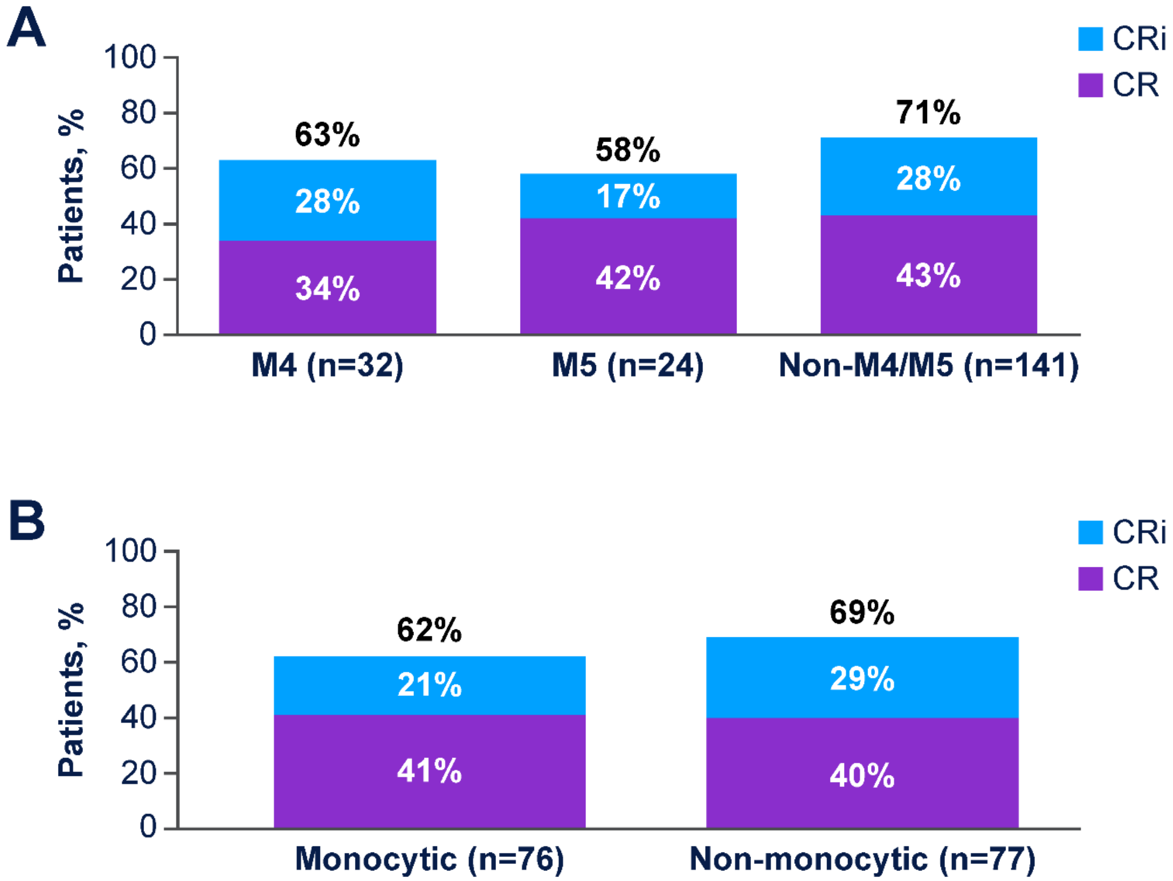

Supplemental Figure 3. Clinical outcomes for patients treated with venetoclax-azacitidine by GEP subtype using the 6-gene panel based on ELN guidelines. (A) OS by GEP subtype. (B) CR + CRi rates by GEP subtype. CR, complete remission; CRi, complete remission with incomplete marrow recovery; GEP, gene expression profiling; mAML, monocytic AML; mOS, median overall survival; OS, overall survival.

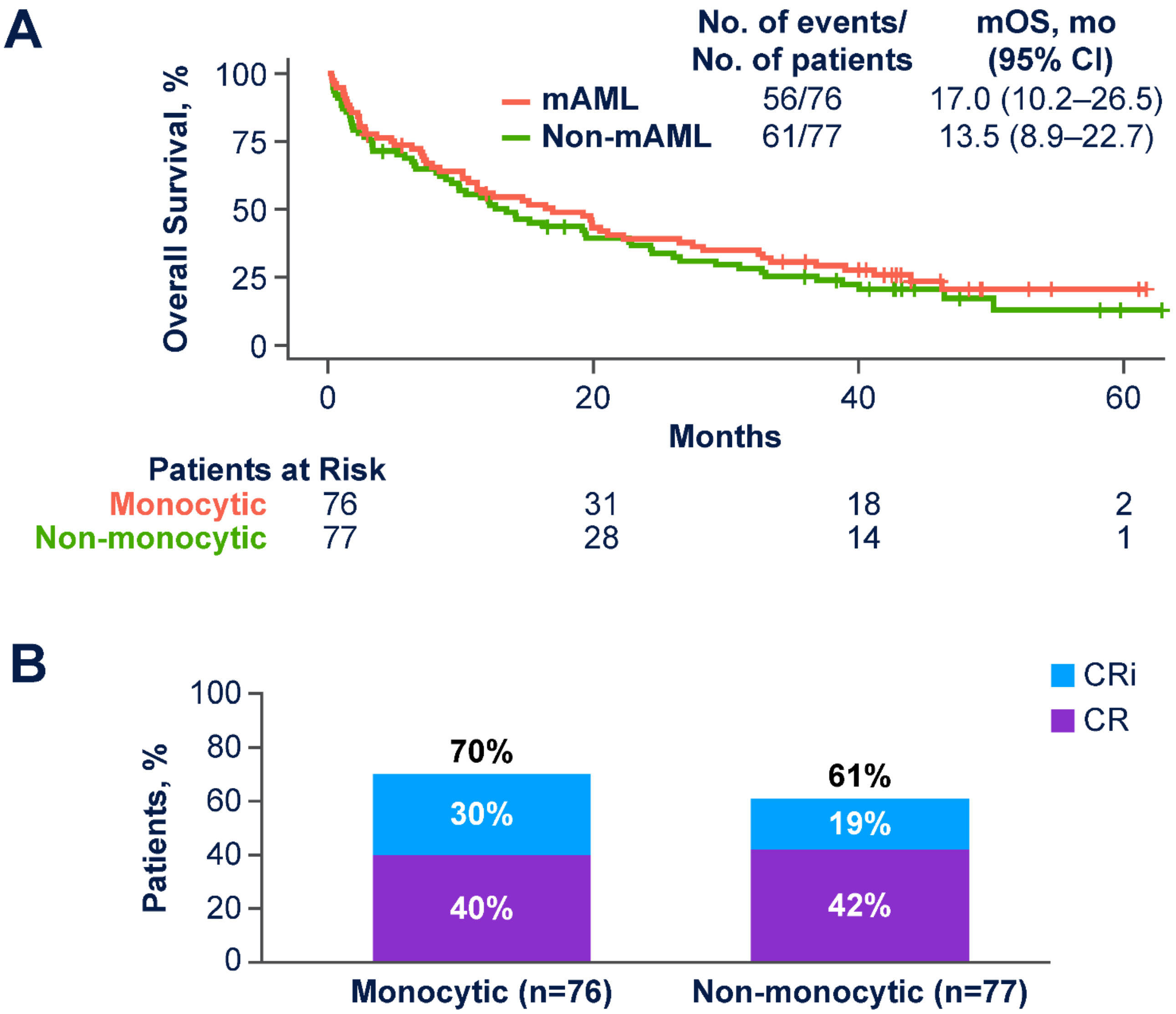

Supplemental Figure 4. OS for patients treated with venetoclax-azacitidine by GEP subtype. Data are presented for patients in the top quartile of the gene expression signature. GEP, gene expression profiling; mAML, monocytic AML; mOS, median overall survival; OS, overall survival.

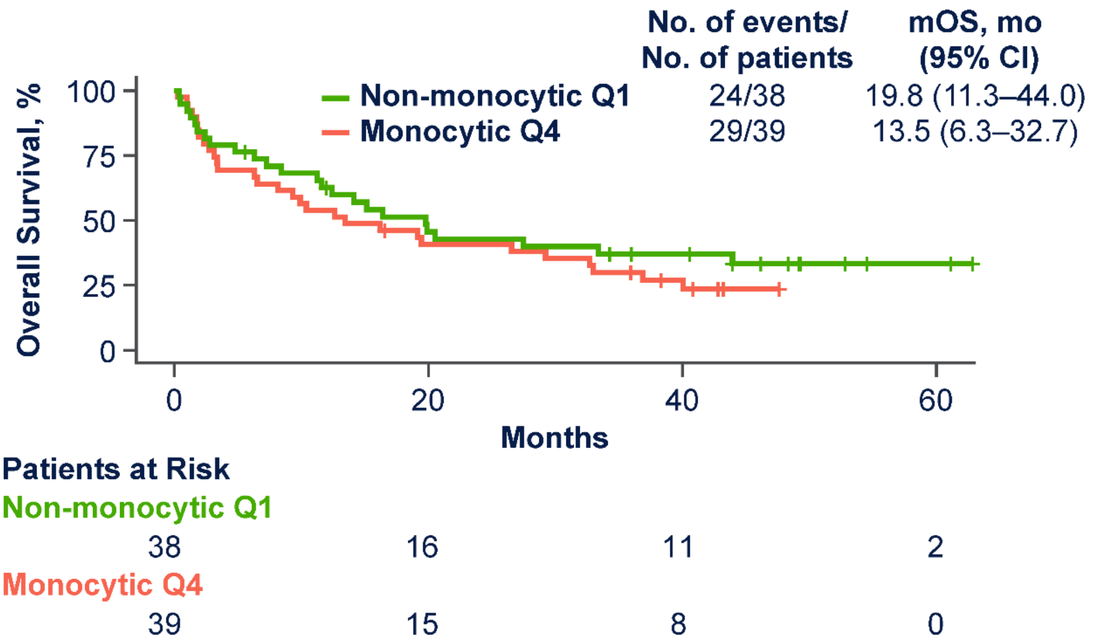

Supplemental Figure 5. OS for patients treated with venetoclax-azacitidine by monocytic vs non-monocytic AML status and (A) by *NPM1* wild-type and (B) *N/KRAS* wild-type. AML, acute myeloid leukemia; mAML, monocytic AML; mOS, median overall survival; mut, mutation; OS, overall survival.

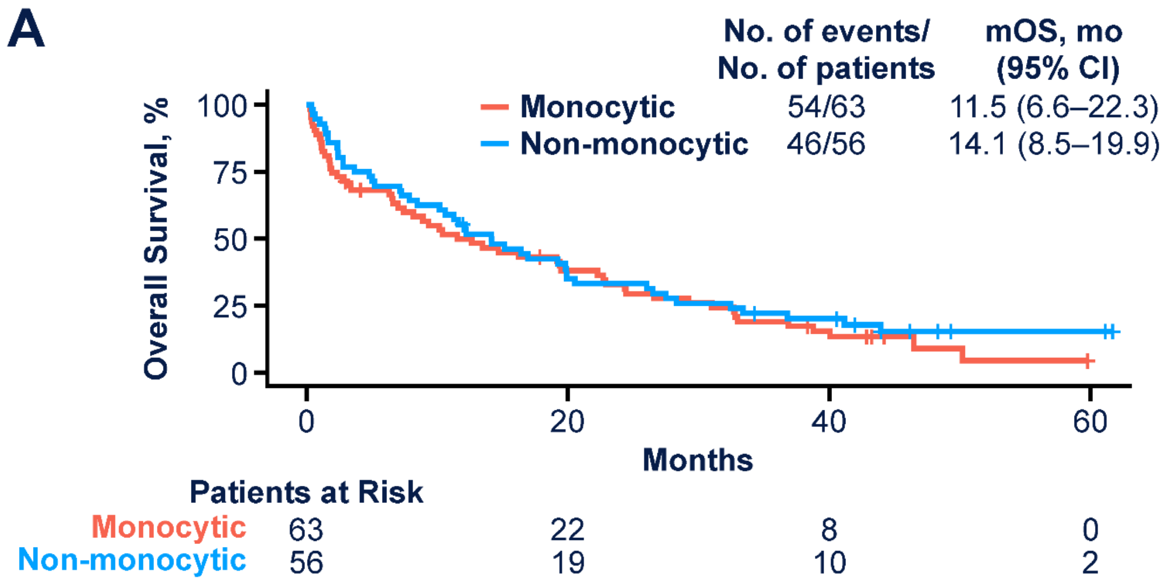

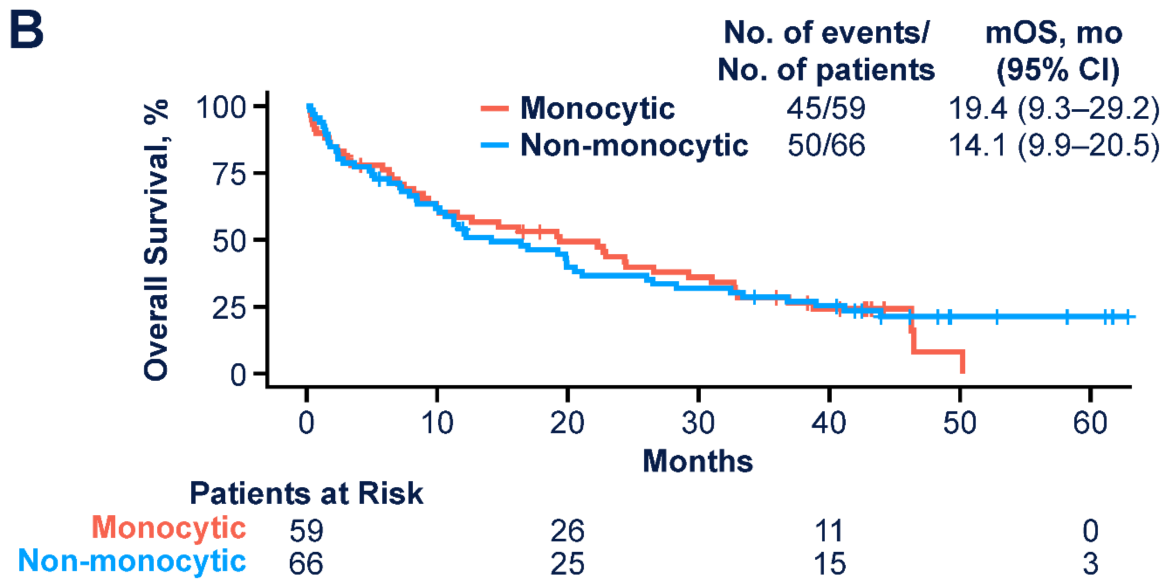

Supplemental Figure 6. **Gene expression by mutation type in monocytic AML vs non-monocytic AML.** AML, acute myeloid leukemia.

A. *BCL2* Gene Expression

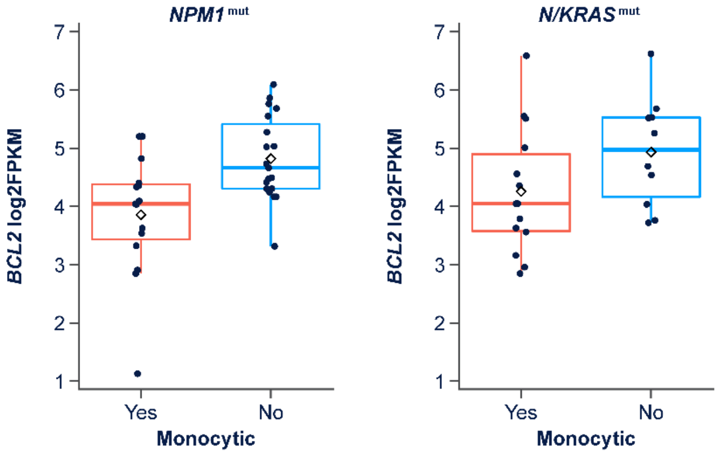

Group	N	*BCL2* log2FPKM
Monocytic	15	4.1
Non-monocytic	19	4.7

Group	N	*BCL2* log2FPKM
Monocytic	14	4.1
Non-monocytic	10	5.0

B. *BCL2A1* Gene Expression

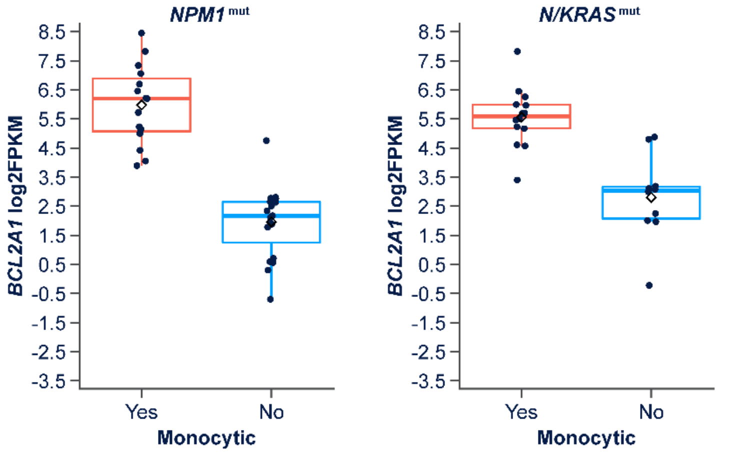

Group	N	*BCL2A1* log2FPKM
Monocytic	15	6.2
Non-monocytic	19	2.2

Group	N	*BCL2A1* log2FPKM
Monocytic	14	5.6
Non-monocytic	10	3.0

C. *BCL2L1* Gene Expression (Encoding BCL-xL)

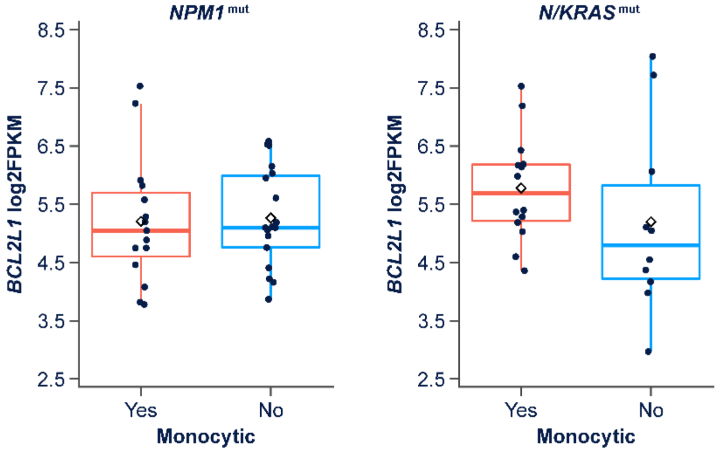

Group	N	*BCL2L1* log2FPKM
Monocytic	15	5.1
Non-monocytic	19	5.1

Group	N	*BCL2L1* log2FPKM
Monocytic	14	5.7
Non-monocytic	10	4.8

D. *MCL1* Gene Expression

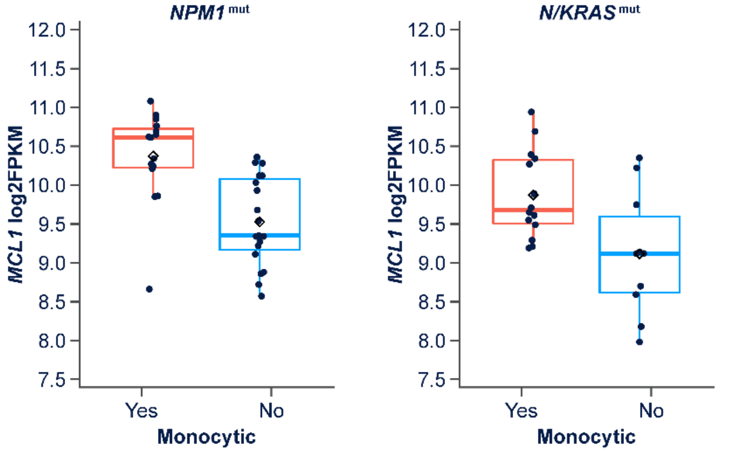

Group	N	*MCL1* log2FPKM
Monocytic	15	10.6
Non-monocytic	19	9.4

Group	N	*MCL1* log2FPKM
Monocytic	14	9.7
Non-monocytic	10	9.1

Supplemental Table 1. Patient characteristics by FAB subtype.

Patient characteristics	M4<br>n=32	M5<br>n=24	Non-M4/M5<br>n=141
**Age, median years (range)**	76.0 (62.0-91.0)	76.0 (67.0-84.0)	77.0 (58.0-90.0)
**Age <75 years, n (%)**	12 (37.5)	9 (37.5)	57 (40.4)
**Age ≥75 years, n (%)**	20 (62.5)	15 (62.5)	84 (59.6)
**Female sex, n (%)**	11 (34.4)	7 (29.2)	55 (39.0)
**Male sex, n (%)**	21 (65.6)	17 (70.8)	86 (61.0)
**Primary AML, n (%)**	18 (56.2)	20 (83.3)	120 (85.1)
**Secondary AML, n (%)**	14 (43.8)	4 (16.7)	21 (14.9)
**Cytogenetic risk = intermediate, n (%)**	20 (62.5)	20 (83.3)	91 (64.5)
**Cytogenetic risk = poor, n (%)**	12 (37.5)	4 (16.7)	50 (35.5)
**ECOG PS <2, n (%)**	21 (65.6)	10 (41.7)	81 (57.4)
**ECOG PS ≥2, n (%)**	11 (34.4)	14 (58.3)	60 (42.6)
**<30% blasts, n (%)**	8 (25.0)	6 (25.0)	45 (31.9)
**≥30% to <50% blasts, n (%)**	5 (15.6)	6 (25.0)	27 (19.1)
**≥50% blasts, n (%)**	19 (59.4)	12 (50.0)	69 (48.9)

Percentages calculated from the total number of patients in FAB classification (M4, M5, or Non-M4/M5) with available data. AML, acute myeloid leukemia; ECOG PS, Eastern Cooperative Oncology Group Performance Status; FAB, French-American-British classification.

Supplemental Table 2. Patient characteristics by GEP-defined monocytic categorization.

Patient characteristics	Monocytic n=76	Non-monocytic n=77
**Age, median years (range)**	76.5 (61.0-91.0)	77.0 (49.0-90.0)
**Age <75 years, n (%)**	27 (35.5)	30 (39.0)
**Age ≥75 years, n (%)**	49 (64.5)	47 (61.0)
**Female sex, n (%)**	28 (36.8)	35 (45.5)
**Male sex, n (%)**	48 (63.2)	42 (54.5)
**Primary AML, n (%)**	56 (73.7)	61 (79.2)
**Secondary AML, n (%)**	20 (26.3)	16 (20.8)
**Cytogenetic risk = intermediate, n (%)**	50 (65.8)	54 (70.1)
**Cytogenetic risk = poor, n (%)**	26 (34.2)	23 (29.9)
**ECOG PS <2, n (%)**	45 (59.2)	46 (59.7)
**ECOG PS ≥2, n (%)**	31 (40.8)	31 (40.3)
**<30% blasts, n (%)**	0	0
**≥30% to <50% blasts, n (%)**	28 (36.8)	19 (24.7)
**≥50% blasts, n (%)**	48 (63.2)	58 (75.3)

Percentages calculated from the total number of patients in GEP classification (monocytic AML or non-monocytic AML) with available data. AML, acute myeloid leukemia; ECOG PS, Eastern Cooperative Oncology Group Performance Status; GEP, gene expression profiling.

Supplemental Table 3. Mutations by FAB subtype.

	NPM1 Mutation, n (%)	N/KRAS Mutation, n (%)
	22/145 (15.2)	24/145 (16.6)
**M4**	3/22 (13.6)	8/24 (33.3)
**M5**	4/22 (18.2)	4/24 (16.7)
**Non-M4/M5**	15/22 (68.2)	12/24 (50.0)

FAB, French-American-British classification.

Supplemental Table 4. **CR + CRi for patients treated with venetoclax-azacitidine by mutations in the GEP-defined subtypes.**

Mutation, n (%)	Monocytic AML, n=74		Non-monocytic AML, n=74	
	n (%)	CR + CRi, % (95% CI)	n (%)	CR + CRi, % (95% CI)
NPM1 ^{mut}	11 (14.9)	63.6 (35.4-84.8)	18 (24.3)	72.2 (49.1-87.5)
NPM1 ^{wt}	63 (85.1)	60.3 (48.0-71.5)	56 (75.7)	66.1 (53.0-77.1)
N/KRAS ^{mut}	15 (20.3)	33.3 (15.2-58.3)	8 (10.8)	75.0 (40.9-92.8)
N/KRAS ^{wt}	59 (79.7)	67.8 (55.1-78.3)	66 (89.2)	66.7 (54.7-76.8)

AML, acute myeloid leukemia; CR, complete remission; CRi, complete remission with incomplete marrow recovery; GEP, gene expression profiling; mut, mutation; wt, wild-type.

Supplemental Table 5. Overlapping mutations.

**Mutation, n (%)**	***NPM1***		***N/KRAS***	
	Monocytic AML (n=11)	Non-monocytic AML (n=18)	Monocytic AML (n=15)	Non-monocytic AML (n=8)
NPM1	-	-	2 (13.3)	2 (25.0)
N/KRAS	2 (18.2)	2 (11.1)	-	-

AML, acute myeloid leukemia.
